# Case Report: Massive Spontaneous Pneumothorax—A Rare Form of Presentation for Severe COVID-19 Pneumonia

**DOI:** 10.3390/medicina57020082

**Published:** 2021-01-20

**Authors:** Adina Maria Marza, Alina Petrica, Florina Nicoleta Buleu, Ovidiu Alexandru Mederle

**Affiliations:** 1 Department of Surgery, Faculty of Medicine, Multidiciplinary Center for Research, Evaluation, Diagnosis and Therapies in Oral Medicine Victor Babes University of Medicine and Pharmacy, 300041 Timisoara, Romania; marza.adina@umft.ro (A.M.M.); mederle.ovidiu@umft.ro (O.A.M.); 2 Emergency Clinical Municipal Hospital, 300041 Timisoara, Romania; 3 Emergency Clinical County Hospital Pius Brînzeu, 300736 Timisoara, Romania; buleu.florina@gmail.com; 4 Department of Cardiology, Faculty of Medicine, Victor Babes University of Medicine and Pharmacy, 300041 Timisoara, Romania

**Keywords:** spontaneous pneumothorax, COVID-19, thoracostomy during pandemia

## Abstract

*Background and Objectives:* Coronavirus disease 2019 (COVID-19), caused by severe acute respiratory syndrome coronavirus 2 (SARS-CoV-2) infection is a viral disease that is spreading worldwide and became a pandemic. Although most of the time, the symptoms of the infection are flu like, a percentage of patients develop severe forms, along with severe complications. Many of them are known among front-line health workers, but the number of uncommon presentations and complications has increased. This case report aims to alert healthcare workers on less common forms of presentation, and to introduce this differential diagnosis in the evaluation of patients with COVID-19, given the increasing occurrence of pneumothorax in patients who are not mechanical ventilated. *Case presentation*: A 57-year-old female patient came to the Emergency Department (ED) by ambulance, with acute respiratory failure. She had SpO_2_ (peripheral O_2_ saturation ) = 43% on room air at home, and 86% on admission in ED after oxygen delivery (on a reservoir mask). SARS-CoV-2 infection was suspected based on symptoms that started three days ago (fever, dry cough, dyspnea, and fatigability). Blood was taken for lab tests, pharyngeal and nasal swabs for the reverse transcription–PCR (RT-PCR) test, and native computed tomography (CT) was scheduled. The thoracic CT scan showed massive right pneumothorax, partially collapsed lung, multiple bilateral lung infiltrates with a ground glass aspect and the RT-PCR test came back positive for SARS-CoV-2 infection. Despite the prompt diagnosis and treatment of pneumothorax (thoracostomy was performed and the drain tube was placed), the patient died after a long hospitalization in the intensive care unit. *Conclusion:* Secondary spontaneous pneumothorax (SSP), as a complication in severe forms of COVID-19 pneumonia, especially in female patients without risk factors is rare, and early diagnosis and treatment are essential for increasing the survival chances of these patients.

## 1. Introduction

Coronavirus disease 2019 (COVID-19) was first reported in Wuhan, Hubei Province, China, in December 2019 [1]. Since its first description, severe acute respiratory syndrome coronavirus 2 (SARS-CoV-2) has led to a pandemic, which was officially declared a global health emergency by the World Health Organization (WHO) on March 11, 2020 [2]. Recent studies have shown that the average age of infected people is around 50 years, even though all ages of the population are susceptible to SARS-CoV-2 infection. However, the clinical manifestations differ according to age [3,4]. A meta-analysis conducted by Peckham et al. which included more than 3 million cases, showed that, although no significant differences were observed between men and women with confirmed COVID-19, male patients were almost three times more likely to be admitted in the intensive care unit due to complications of the disease and a higher risk of death compared to women [5]. The symptoms of SARS-CoV-2 infection have been widely characterized in large studies, with fever, cough, and dyspnea being the most frequent [4,6]. A percentage of patients develop severe forms, along with severe complications.

Spontaneous pneumothorax (SP) is a rare complication of COVID-19 pneumonia, with an incidence of less than 1‰ according to the current literature [7], suggesting that pneumothorax is either uncommon or underreported in patients with COVID-19 [8]. Several case reports have shown that SP associated with COVID-19 pneumonia led to a higher severity and fatal outcome [9]. The prognosis of these patients might be related to the severity of lung lesions, although there is no clear correlation with the magnitude of SP [10]. The timing of SP in the evolution of COVID-19 pneumonia is uncertain. In a recent review of the current literature conducted by Dennison et al. on 32 case reports of spontaneous pneumothorax or pneumomediastinum out of 58 COVID-19 pneumonia infections, SP occurred days to weeks after the onset of symptoms [11]. Therefore, issues like risk factors, timing, outcome, still need to be thoroughly researched.

Here, we describe a case of a 57-year-old woman, who had no prior lung injury or other risk factors for spontaneous pneumothorax, was never a smoker, and was not mechanically ventilated, who developed massive spontaneous pneumothorax after only 3 days of symptoms of SARS-CoV-2 infection.

## 2. Case Presentation

A 57-years-old woman with a history of essential hypertension was brought into the emergency department (ED) with acute respiratory failure by an ambulance with a doctor. At home, she was found with a significantly decreased peripheral O_2_ saturation (SpO_2_) of 43% while breathing room air, saturation that increased to 86% after oxygen delivery (on a reservoir mask). On physical examination the patient presented tachypnea (34 breaths per minute), pale sweaty skin, hemodynamically stable, blood pressure 127/66 mmHg, a heart rate of 109 beats/min, a body temperature of 37.8 °C, Glasgow Coma Score (GCS) = 15/15. The patient complained of fever, dry cough, dyspnea on exertion and fatigue, that started 3 days before the presentation, claiming that, during the previous night, the dyspnea suddenly worsened and an anterior chest pain appeared. She had no other comorbidities except essential hypertension under treatment. Based on the history and clinical examination, SARS-CoV-2 infection was suspected. Prone position was initiated in order to improve ventilation, which the patient did not tolerate, so she returned to the seated position. She received an intravenous perfusion with 1 g acetaminophen, 8 mg dexamethasone, 1500 mg vitamin C, 40 mg pantoprazole and 500 mL normal saline (0.9%) solution. Blood was taken for lab tests and she was scheduled for a thoracic CT (computed tomography) scan. The pharyngeal and nasal swabs were taken for the RT-PCR test, which came positive after several hours.

**Figure 1:**
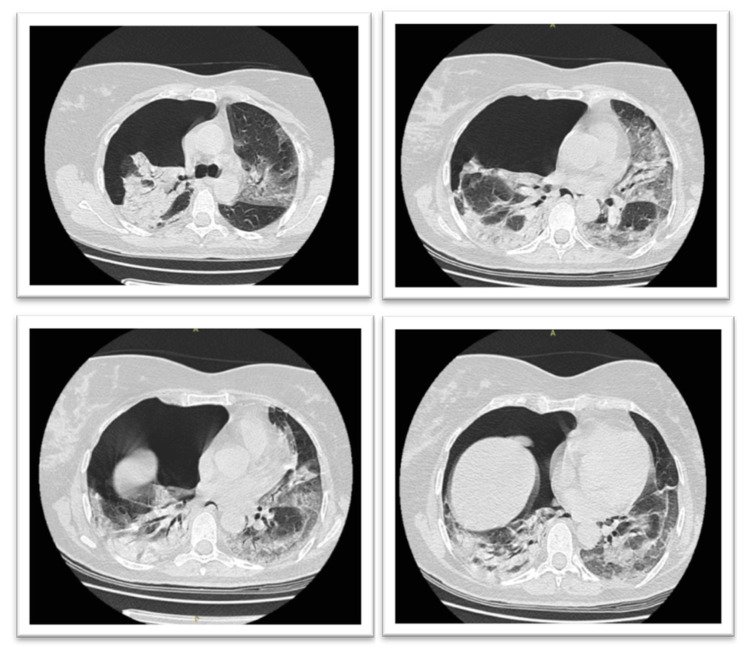
CT scan at admission in ED: massive right pneumothorax, partially collapsed lung, slightly left-displaced heart, multiple bilateral ground glass lung infiltrates.

The thoracic CT scan showed massive right pneumothorax (90 mm), lung partially collapsed, slightly left-displaced heart, multiple bilateral lung infiltrates with a ground-glass aspect that occupied about 65% of lung fields—CO-RADS classification 5, typical COVID-19 aspect. (Figure 1)

The thoracostomy was performed and the drain tube was placed in the right fifth intercostal space, the medium axillary line, under local anesthesia. The patient’s condition partially improved and oxygen saturation increased by approximately 6% (SpO_2_ = 92–93%) 15–20 min after the drain tube was placed. Oxygen therapy on a reservoir mask with 15 L/min was continued, and remdesivir 200 mg as loading dose was initiated, then 100 mg at 24 h, lopinavir/ritonavir 200 mg/50 mg—2 pills every 12 h, enoxaparin 60 mg twice daily, dexamethasone 8 mg three times daily, pantoprazole 40 mg twice daily, vitamin C 1 g every 6 h, Ceftriaxone 1 g twice daily, her previous medicines for hypertension, acetaminophen on need and soluble regular insulin according to her glycemia level (given the constant high values observed during her hospitalization), vitamin D, B1, B6, zinc and alprazolam.

Laboratory test results found to be pathological are shown in Table 1.

**Figure 2:**
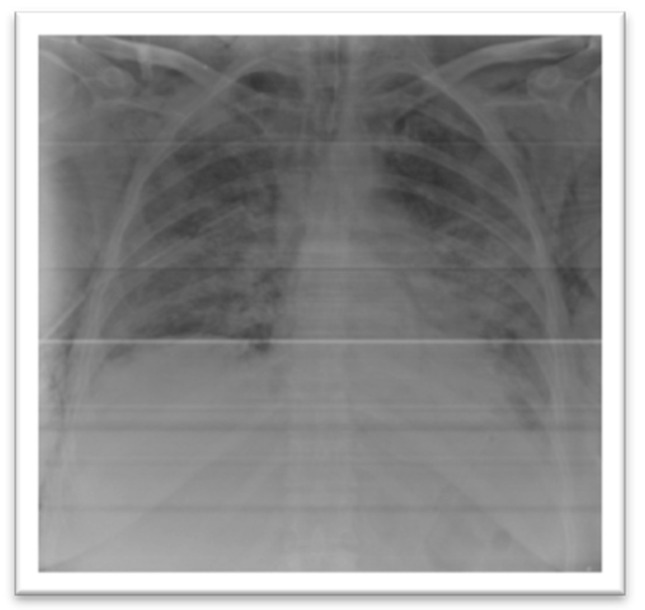
Chest radiography of the patient the day after insertion of the drain tube, demonstrating the re-expansion of the lung and multiple bilateral opacities in the context of COVID-19 infection.

After a few hours, a control chest X-ray was performed, confirming the correct placement of the drain tube with full expansion of the collapsed lung (Figure 2). During the day, the patient’s breathing worsened (tachypnea 40 breaths/minute) with increased breathing effort, decreased oxygen saturation to 70%, hypoxemia (pO2 = 36 mmHg on arterial blood gases (ABG)) despite maximum oxygen delivery so she was admitted to the intensive care unit and non-invasive ventilation (NIV) was initiated. The patient tolerated non-invasive ventilation for a few hours, but then the condition of the patient worsened again, and the endotracheal intubation and invasive mechanical ventilation were decided.

During hospitalization in the intensive care unit, the patient had a fluctuating evolution, and despite the treatment (medication, kinetotherapy, prone position, hydro-electrolyte rebalance solutions), the patient’s condition was deteriorating and required vasopressor support and mechanical ventilation. At 19 days after admission in the intensive care unit, the patient had cardiac arrest through asystole and did not respond to resuscitation maneuvers.

## 3. Discussion

Spontaneous pneumothorax is a type of pneumothorax that develops in the absence of trauma [12]. It is classified as primary and secondary SP. Primary spontaneous pneumothorax (PSP) occurs in patients without pre-existing lung disease, compared with secondary spontaneous pneumothorax (SSP), which is a complication occurring in an affected lung. PSP is common in young adults, with a higher incidence in men than in women (7.4 to 18 per 100,000 men and 1.2 to 6 per 100,000 women) [13].

Risk factors for PSP include male gender, tall and thin stature and smoking [14]. In SSP, the most common underlying disorders are COPD with a predominance of pulmonary emphysema, cystic fibrosis, tuberculosis, lung cancer, interstitial pneumonitis, and Pneumocystis carinii pneumonia associated with human immunodeficiency virus. While PSP usually occurs between the ages of 10 and 30, the maximum incidence of SSP is observed in the following years—between the ages of 60 and 64—depending on the baseline condition [12]. Identifying the cause of an SSP is crucial, as the immediate and long-term management of the SSP differs from that of the PSP, along with significantly more serious consequences [15].

Massive pneumothorax is a major, life-threatening emergency that must be identified and treated very promptly [16], regardless if the patient presents to ED or it occurs as a complication in COVID 19 ward patients. Our patient had a massive pneumothorax that was timely solved, but still she had a fatal outcome. However, we cannot state that the size of the pneumothorax had a defining role in the patient’s evolution as there are studies with COVID-19 patients experiencing small pneumothorax and still a poor prognosis [9,10].

Further research is needed in order to identify other possible factors influencing the evolution of patients with COVID-19 pneumonia and SP. In a large study by Òscar Miró et al., the most frequent met comorbidities in patients with COVID-19 and SP were hypertension (37.5% of cases), asthma (20% of cases) and diabetes (17.5% of cases) [7]. Our patient had both hypertension and diabetes (not previously known); however, we cannot state a direct relation between these factors and SP development. Nevertheless, these comorbidities and the lesions’ extension were determinant for the patient’s outcome.

Spontaneous pneumothorax is a commonly known complication in patients with acute respiratory distress syndrome (ARDS), where the most frequent causes are pressure and volume-related alveolar rupture [17]. Histological examination of lung biopsy samples in a patient who died from COVID-19 pneumonia showed desquamation of pneumocytes and hyaline membrane formation, indicating ARDS [15].

We suspect that our patient developed SSP because of lung lesions caused by COVID-19 infection, given the fact that she was not a smoker and not known to have other risk factors. The patient had sudden onset dyspnea, chest pain, tachycardia, hypoxia, and increased D-Dimers, so one of the considered differential diagnosis was pulmonary embolism. However, it was initially decided to perform a native thoracic CT scan, which led to the correct diagnosis and completely changed the patient’s therapeutic management, being the first patient with such a complication of SARS-CoV-2 infection in our clinic. As opposed to different case reports from the literature, where the CT scans were performed late in the course of patients’ evolution and dictated by the deterioration of the patient, in our case, the CT scan was performed from the admittance to the ED [9,11]. The large availability of CT scan in our hospital helped us to better quantify the overall severity of patients with COVID-19 pneumonia and led to a prompt management of our case.

Despite the timely diagnosis and treatment of SSP, the patient’s evolution was marked by severe lung damage from COVID-19 pneumonia, and after a long hospitalization in the intensive care unit, the patient died, emphasizing the importance of the underlying lung disease in spontaneous secondary pneumothorax. Patients with COVID-19 pneumonia and SP should be carefully monitored to prevent respiratory deterioration, no matter the size of the pneumothorax [10].

## 4. Conclusions

Secondary spontaneous pneumothorax should always be considered as a differential diagnosis in the assessment of patients with SARS-CoV-2 infection and acute respiratory failure. Prompt diagnosis and treatment are crucial in the further evolution of the patient. Large studies are needed to measure the prognosis of these pulmonary complications in patients with SARS-CoV-2 infection, but since the cases are sporadic, any detailed report may have an added value.

## Figures and Tables

**Table 1:** Pathological values of the laboratory tests performed in the Emergency Department.

Laboratory Test	Conventional Units	Value	Reference Range Value
WBC	×10^9^/µL	3.8	4–10
Lymphocytes	×10^3^/µL	0.7	1–3
AST	U/L	107	2–32
ALT	U/L	80	2–33
Ferritin	ng/mL	1463	15–150
Blood glucose	mg/dL	361	74–106
CRP	mg/dL	85.9	0–5
D-dimers	ng/mL	1212.8	age × 10
aPTT	seconds	extremely low *	25–36

WBC = white blood cells; AST = aspartate aminotransferase; ALT = alanine aminotransferase; CRP = C-reactive protein; aPTT = activated partial thromboplastin time. * value under the detection limit of the equipment.

## Data Availability

The data presented in this study are available on request from the corresponding author. The data are not publicly available due to patient confidentiality.
